# Initial Incomplete Thermal Ablation Is Associated With a High Risk of Tumor Progression in Patients With Hepatocellular Carcinoma

**DOI:** 10.3389/fonc.2021.760173

**Published:** 2021-10-18

**Authors:** Jie Tan, Tian Tang, Wei Zhao, Zi-Shu Zhang, Yu-Dong Xiao

**Affiliations:** ^1^ Department of Radiology, Second Xiangya Hospital, Central South University, Changsha, China; ^2^ Department of Interventional Radiology, the Affiliated Cancer Hospital of Xiangya School of Medicine, Central South University, Changsha, China

**Keywords:** thermal ablation, therapeutic outcome, survival, transarterial chemoembolization, propensity score-matching

## Abstract

**Purpose:**

To investigate whether incomplete thermal ablation is associated with a high risk of tumor progression in patients with hepatocellular carcinoma (HCC), and to compare the efficacy of repeated thermal ablation and transarterial chemoembolization (TACE) for residual tumor after incomplete ablation.

**Methods:**

This retrospective study included 284 patients with unresectable HCC who underwent thermal ablation from June 2014 to September 2020. The response of the initially attempted ablation was classified into complete (n=236) and incomplete (n=48). The progression-free survival (PFS) and overall survival (OS) were compared between patients with complete and incomplete responses, before and after a one-to-one propensity score-matching (PSM), and between patients in whom repeated ablation or TACE was performed after a first attempt incomplete ablation.

**Results:**

After PSM of the 284 patients, 46 pairs of patients were matched. The PFS was significantly higher in the complete response group than in the incomplete response group (P<0.001). No difference in OS was noted between two groups (P=0.181). After a first attempt incomplete ablation, 29 and 19 patients underwent repeated ablation and TACE, respectively. There were no significant differences in PFS (P=0.424) and OS (P=0.178) between patients who underwent repeated ablation and TACE. In multivariate Cox regression analysis, incomplete response (P<0.001) and Child-Pugh class B (P=0.017) were independent risk factors for tumor progression, while higher AFP level (P=0.011) and Child-Pugh class B (P=0.026) were independent risk factors for poor OS.

**Conclusion:**

Although patients with incomplete ablation are associated with tumor progression compared with those with complete ablation, their OS is not affected by incomplete ablation. When patients present with residual tumors, TACE may be an alternative if repeated ablation is infeasible.

## Introduction

Hepatocellular carcinoma (HCC) accounts for 90% of primary liver cancer, which is the fourth most common cause of cancer-related deaths worldwide. According to several guidelines, thermal ablation is recommended as a curative therapy for patients with early-stage hepatocellular carcinoma (HCC) who are not candidates for surgical resection and liver transplantation (1–3). However, due to its insufficient and disproportionate ablative zone, thermal ablation is constricted in lesions of a large size or in unfavorable locations (4–6). As previous studies reported, the insufficient ablation rate after thermal ablation is as high as 55% to 77% in tumors larger than 3 cm (7, 8). In several pre-clinical studies, the relationship between insufficient tumor ablation-induced reversible cellular injury and tumor progression has been demonstrated (9, 10). However, whether incomplete tumor ablation is associated with tumor progression in patients with HCC has not been elucidated in clinical study. Furthermore, the standard therapy for HCC patients with residual tumor after a first attempt incomplete thermal ablation has not yet been established. Therefore, the present study aims to investigate whether incomplete thermal ablation is associated with tumor progression and to compare the treatment efficacy of repeated thermal ablation and transarterial chemoembolization (TACE) in patients with a residual tumor after a previously attempted thermal ablation.

## Materials and Methods

### Patients

This retrospective study was approved by the institutional review boards of the Second Xiangya Hospital and the Affiliated Cancer Hospital of Xiangya School of Medicine and was performed in accordance with the Declaration of Helsinki. The need for written informed consent was waived by the institutional review board due to the retrospective nature of the present study.

**Figure 1:**
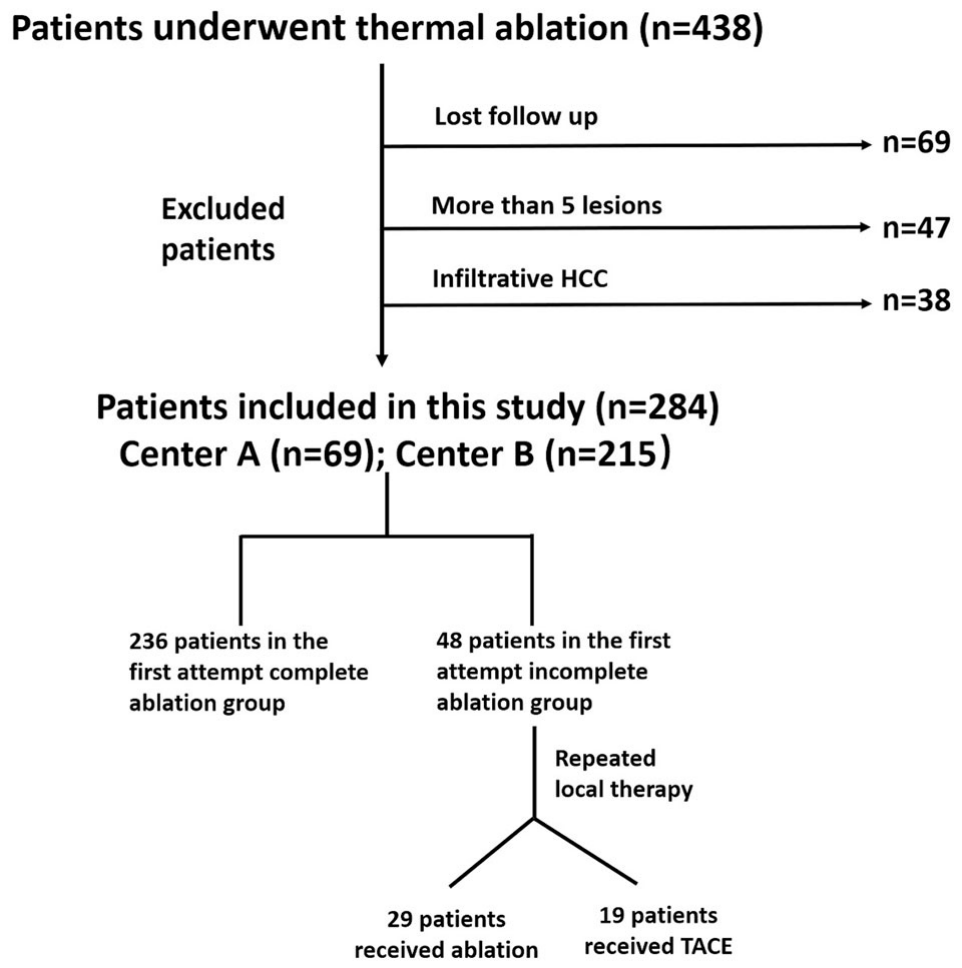
Flowchart of the study population.

Between June 2014 and September 2020, 438 consecutive patients with HCC, who underwent thermal ablation as an initial therapy at two institutions, were selected for this study. Patients meeting the following criteria were included: those with (a) a preserved liver function (Child-Pugh A or B); (b) an Eastern Cooperative Oncology Group performance status 0; (c) at least one target lesion for measurement; and (d) the absence of macrovascular invasion and extrahepatic metastasis. The exclusion criteria were as follows: patients who (a) did not follow-up (n=69); (b) had more than five HCC lesions (n=47); (c) had infiltrative HCC (n=38). Finally, 284 patients were included in the present study. Among those 284 patients, 69 were from institution A and 215 were from institution B. The flowchart of the study population is shown in **Figure 1**.

### Thermal Ablation Procedure

Thermal ablation was performed under computed tomography (CT) or ultrasound guidance using a microwave ablation (MWA) (KY-2000, Kangyou Medical Instrument Co. Ltd., China) or a radiofrequency ablation (RFA) system (CELON Power System, Olympus Corporation, Japan) by four board-certified senior interventional radiologists. The microwave ablation was set at 60 to 80 W for 3 to 20 min. The radiofrequency ablation was set at 30 to 80 W for 10 to 40 min. For tumors larger than 3.0 cm, an overlapping ablation was performed. Contrast-enhanced CT immediately after thermal ablation was routinely performed to assure a minimum safety margin of 0.5 cm when possible. For multifocal lesions, a maximum of three lesions was ablated in a single session, and the remaining were ablated within one week after the first session.

Contrasted-enhanced CT or magnetic resonance (MR) was routinely performed approximately four to six weeks after the first attempt thermal ablation. The treatment response was classified into complete response and incomplete response according to the modified Response Evaluation Criteria in Solid Tumors (mRECIST). An incomplete response was defined as residual viable tumor foci at the edge of the ablative zone while a complete response was defined as an ablated lesion with no viable tumor. Patients with multiple tumors were classified into the complete response group only if all the tumors were ablated completely in the first attempt itself; otherwise, they were categorized into the incomplete response group.

### Repeated Thermal Ablation and TACE

In patients with an incomplete response after a first attempt ablation, the feasibility of repeated thermal ablation was assessed. Usually, the lesion was considered infeasible to ablation when it was in an unfavorable location or had poor conspicuity on unenhanced CT or ultrasound. When considered feasible, the patients were treated with repeated thermal ablation. When considered infeasible, TACE was performed as an alternative therapeutic option by several board-certified senior interventional radiologists. The femoral artery was routinely catheterized. Selective hepatic arteriography was performed and a 2.2-F coaxial microcatheter (Carnelian, Tokai Medical Products, Japan) was placed in the tumor feeders. Chemoembolization was performed using a super-selective approach by slowly injecting an oil-epirubicin emulsion. The gel-foam slurry was used to embolize the proximal tumor feeders. The oil-epirubicin emulsion was prepared by mixing iodized oil (up to 15 mL; Lipiodol, Guerbet Asia Pacific Ltd, Hong Kong, China) with distilled water containing 50 mg to 120 mg of epirubicin dissolved in a ratio of 3:1.

### Follow-up

After the procedure, the patients were followed up at 1, 3, 6, and 12 months in the first year and every 6 months subsequently if no recurrence was detected. At each follow-up visit, contrast-enhanced CT/MRI of the liver was performed to evaluate the radiological response according to the mRECIST. Local tumor progression (LTP), intrahepatic distal recurrence (IDR), and extrahepatic recurrence (ER) were recorded. If tumor progression occurred, the choice of treatment for recurrent HCC was dependent on the site of the tumor, liver function, and the general condition of the patient.

The primary outcome was progression-free survival (PFS), which was defined as the time from the first thermal ablation until the date of radiological progression or the last follow-up (April 30, 2021). Radiological progression was defined as patients with LTP, IDR, and ER. The secondary outcome was overall survival (OS), which was defined as the time interval between the first ablation and death from any cause or the last follow-up (April 30, 2021).

### Data Collection

Data on the potential risk factors for PFS and OS were collected, including the first attempt ablation (complete/incomplete), Child-Pugh class (A/B), age (≤56 years/>56 years), gender (male/female), number of tumors (single/multiple), modality of guidance for ablation (CT/ultrasound), type of thermal ablation (MWA/RFA), tumor size (≤3 cm/>3 cm), etiology of hepatitis (none/hepatitis B virus/hepatitis C virus/alcoholic), cirrhosis (presence/absence), level of alpha-fetoprotein (AFP; ≤400 ng/mL/>400 ng/mL), and level of the systemic immune inflammation index (SII; ≤330/>330). The SII was calculated as (N*P)/L, where “N” represents the neutrophil count, “P”, the platelet count, and “L”, the lymphocyte count; a cut-off of 330 for SII was used as previously described (11). In the present cohort, patients were stratified based on the median age (56 years).

### Statistical Analysis

Categorical variables were compared using the Chi-square test or Fisher’s exact test, as appropriate. Continuous variables were compared using the Mann-Whitney U test or t-test, as appropriate. To diminish potential confounding and selection bias in the two groups, propensity score-matching (PSM) was applied. PSM is a statistical method which can reduce the bias due to confounding variables by accounting and matching the covariates that may affect the treatment outcome. Each group was matched according to the generated PSM using a caliper width of 0.1. In patients with multiple lesions, the characteristics of the largest lesion were recorded for analysis. The potential risk factors for PFS and OS were compared using a log-rank test before and after PSM. Cox proportional hazard ratios (HRs) were calculated to identify the independent risk factors for PFS and OS. All potential variables were included in the multivariate Cox regression analysis. Statistical analysis was carried out using a statistical software (Statistical Package for the Social Sciences version 24, International Business Machines Corporation, USA) or R software (version 4.0.2, http://www.R-project.org). P <0.05 was considered to be statistically significant.

## Results

### Baseline Patient Characteristics

The entire study population included 238 males and 46 females, with a mean age of 56.0 ± 11.0 years, ranging from 27 to 81 years. There were 240 patients with hepatitis B, 14 with hepatitis C, and 4 with alcoholic hepatitis. The diagnosis of HCC was based on either histological evidence (n=41) or radiological evaluation with reference to the 2018 version of Liver Imaging Reporting and Data System (n=243). There were 244 patients with Child-Pugh A and 40 patients with Child-Pugh B. In the entire study population, 48 patients were in the very early stage (Barcelona Clinic Liver Cancer [BCLC]-0), 190 were in the early stage (BCLC-A), and 46 were in the intermediate stage (BCLC-B). The baseline characteristics of the patients from the two institutions are shown in **Table 1**.

**Table 1:** The baseline characteristics of patients among the two institutions.

	Center A (n = 69)	Center B (n = 215)	P
Age (Median, IQR)	57 (15)	56 (15)	0.626
Gender			0.757
Male	57	181	
Female	12	34	
First attempt ablation			0.218
Complete response	54	182	
Incomplete response	15	33	
Number of tumors			0.357
Single	50	143	
Multiple	19	72	
Modality of guidance for ablation			<0.001
CT	69	124	
US	0	91	
Type of ablation			<0.001
MWA	43	211	
RFA	26	4	
Tumor size			0.189
≤3cm	30	113	
>3cm	39	102	
Etiology of hepatitis			<0.001
None	16	10	
HBV	52	188	
HCV	1	13	
Alcohol	0	4	
Cirrhosis			0.001
Presence	56	129	
Absence	13	86	
AFP			0.754
≤400 ng/ml	53	169	
>400 ng/ml	16	46	
Child-Pugh class			0.494
A	61	183	
B	8	32	
Platelet (Median, IQR)	107 (86)	119 (77)	0.112
Neutrophil (Median, IQR)	2.86 (1.88)	2.64 (1.59)	0.107
Lymphocyte (Median, IQR)	1.45 (0.83)	1.36 (0.74)	0.586
SII			0.723
≤330	50	151	
>330	19	64	

IQR, interquartile range; CT, computed tomography; US, ultrasound; MWA, microwave ablation; RFA, radiofrequency ablation; HBV, hepatitis B virus; HCV, hepatitis C virus; AFP, alpha fetoprotein; SII, systemic immune inflammation index.

### Comparison of PFS and OS Between the First Attempt Complete Ablation and Incomplete Ablation Before and After PSM

**Figure 2:**
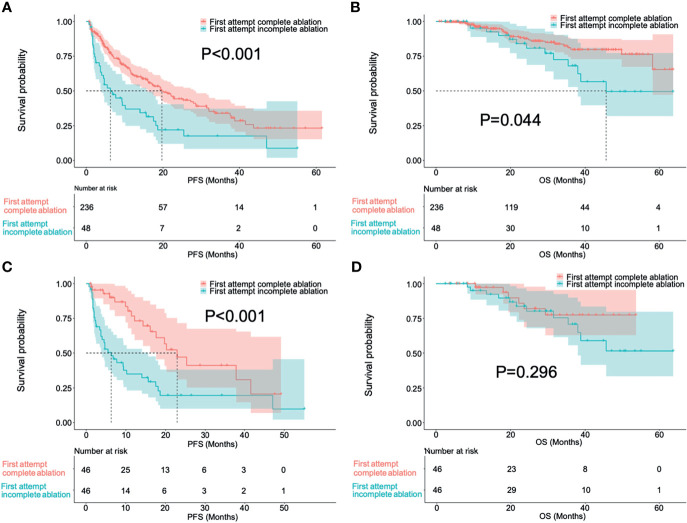
Survival curves in progress-free survival **(A, C)** and overall survival **(B, D)** of patients with first attempt complete ablation and incomplete ablation before and after a one-to-one propensity score-matching.

There were 236 patients in the first attempt complete response group and 48 in the incomplete response group before PSM. The PFS (19.7 months, 95% CI: 15.1-24.3 months vs. 6.3 months, 95% CI: 3.0-9.6 months; P<0.001) and OS (P=0.044) were significantly higher in the complete response group than in the incomplete response group. After a one-to-one PSM analysis, there were 46 pairs of matched patients. The baseline characteristics of the patients before and after PSM are presented in **Table 2**. The PFS was significantly higher in the first attempt complete response group than in the incomplete response group (22.9 months, 95% CI: 15.8-30.0 months vs. 6.3 months, 95% CI: 3.0-9.6 months; P<0.001). However, there was no difference in the OS between the two groups after PSM analysis (P=0.296). **Figure 2** shows the survival curves of PFS and OS of the first attempt complete response and incomplete response groups before and after PSM.

**Table 2:** Patients’ baseline characteristics before and after PSM.

	Before PSM			After PSM		
	First attempt complete ablation (n = 236)	First attempt incomplete ablation (n = 48)	P	First attempt complete ablation (n = 46)	First attempt incomplete ablation (n = 46)	P
Age			0.492			0.404
≤56 years	121	22		25	21	
>56 years	115	26		21	25	
Gender			0.339			0.788
Male	200	38		37	38	
Female	36	10		9	8	
Number of tumors			0.833			0.656
Single	161	32		32	30	
Multiple	75	16		14	16	
Modality of guidance for ablation			0.897			0.822
CT	160	33		32	31	
US	76	15		14	15	
Type of ablation			0.131			0.293
MWA	214	40		35	39	
RFA	22	8		11	7	
Tumor size			<0.001			0.064
≤3cm	134	9		17	9	
>3cm	102	39		29	37	
Etiology of hepatitis			0.180			0.408
None	18	8		7	7	
HBV	202	38		33	37	
HCV	13	1		5	1	
Alcohol	3	1		1	1	
Cirrhosis			0.037			0.834
Presence	160	25		24	25	
Absence	76	23		22	21	
AFP			0.034			0.482
≤400 ng/ml	190	32		35	32	
>400 ng/ml	46	16		11	14	
Child-Pugh class			0.423			1.000
A	201	43		41	41	
B	35	5		5	5	
SII			0.006			0.393
≤330	175	26		30	26	
>330	61	22		16	20	

PSM, propensity score-matching; CT, computed tomography; US, ultrasound; MWA, microwave ablation; RFA, radiofrequency ablation; HBV, hepatitis B virus; HCV, hepatitis C virus; AFP, alpha fetoprotein; SII, systemic immune inflammation index.

### Comparison of PFS and OS Between Repeated Ablation and TACE After First Attempt Incomplete Ablation

**Figure 3:**
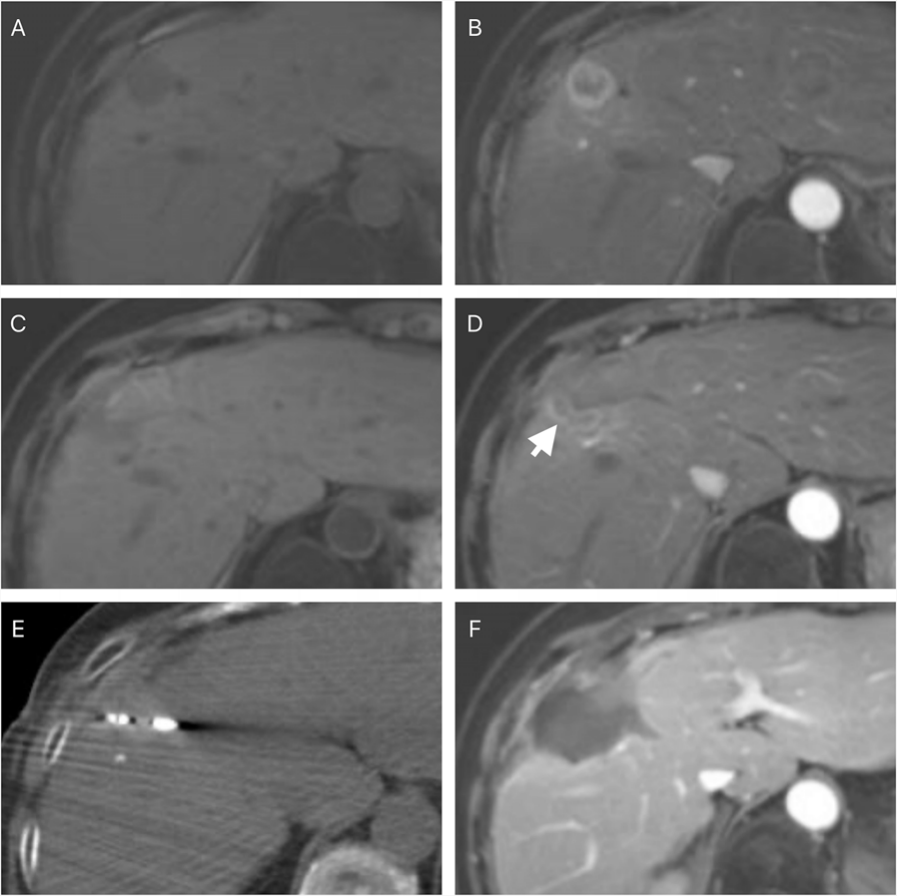
A 62-year-old female with a histopathological proved hepatocellular carcinoma. The pre-ablation magnetic resonance imaging showed the lesion was in the right lobe of the liver **(A)** and a rim enhancement in arterial phase **(B)**. The follow-up magnetic resonance imaging was performed four weeks after the ablation and showed residual tumor foci at the edge of the ablative zone **(C, D)** (white arrow). A repeated ablation was performed **(E)**. Follow-up magnetic resonance imaging was performed four weeks after the ablation and showed a complete response **(F)**.

**Figure 4:**
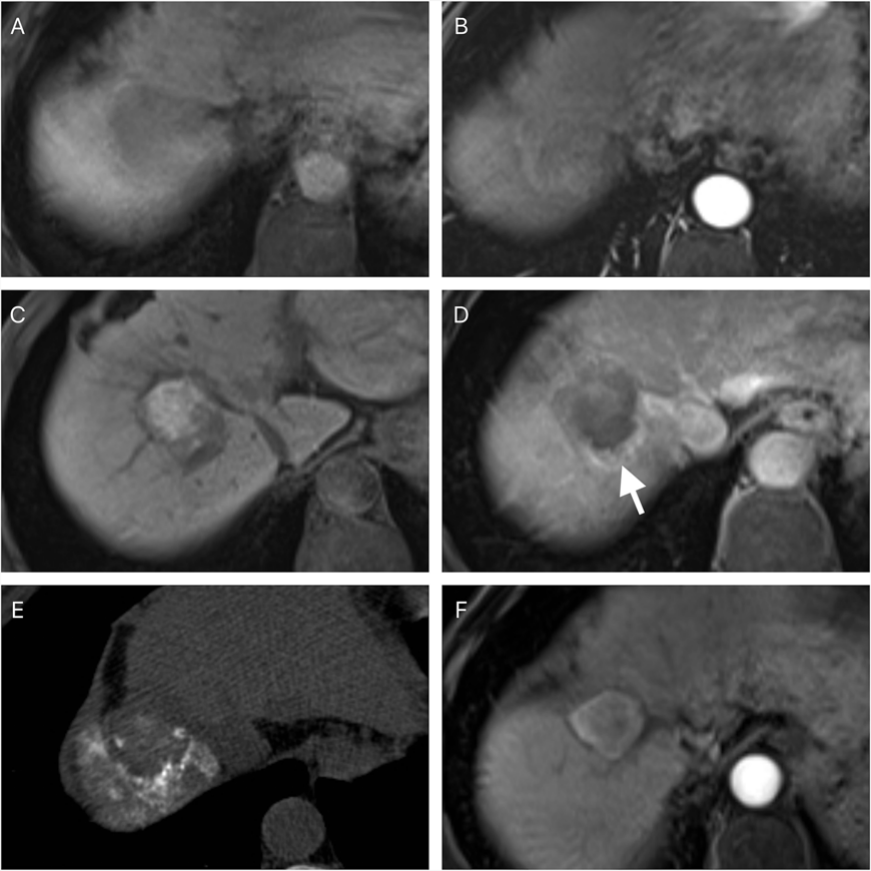
A 56-year-old male with an imaging diagnosed hepatocellular carcinoma. The pre-ablation magnetic resonance imaging showed the lesion was in the right lobe of the liver **(A)** and a non-rim hyperenhancement in arterial phase **(B)**. The follow-up magnetic resonance imaging was performed four weeks after the ablation and showed residual tumor foci at the edge of the ablative zone **(C, D)** (white arrow). A transarterial chemoembolization was performed and iodized oil retention was noted **(E)**. Follow-up magnetic resonance imaging was performed four weeks after the transarterial chemoembolization and showed a complete response **(F)**.

**Figure 5:**
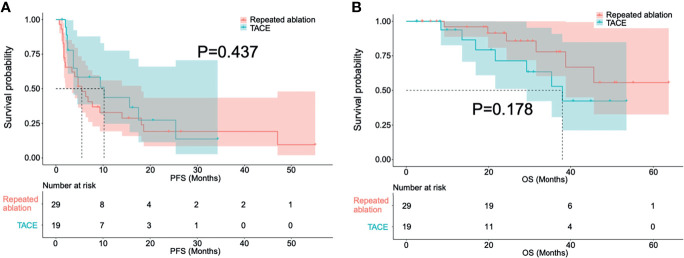
Survival curves in progress-free survival **(A)** and overall survival **(B)** of patients treated with repeated ablation and transarterial chemoembolization after a first attempt incomplete ablation.

After a first attempt incomplete ablation, 29 patients received repeated ablation while 19 received TACE. **Figures 3** and **4** respectively show the typical imaging of patients who received repeated ablation and TACE. In some patients, repeated ablation was not feasible due to the unfavorable location of the lesions (n=13) or poor lesion conspicuity on unenhanced CT or ultrasound (n=6). There were no significant differences in PFS (P=0.437) and OS (P=0.178) between patients with repeated ablation and TACE after a first attempt incomplete ablation. **Figure 5** shows the survival curves of PFS and OS of the repeated ablation and TACE groups after a first attempt incomplete ablation.

### Univariate and Multivariate Analysis for PFS and OS

The univariate analysis for PFS showed that a first attempt incomplete response (P<0.001) was associated with tumor progression. However, between the two groups, no differences in PFS were noted in age (P=0.914), gender (P=0.624), the number of tumors (P=0.507), modality of guidance for ablation (P=0.361), thermal ablation type (P=0.244), tumor size (P=0.560), the etiologies of hepatitis (P=0.834), presence or absence of cirrhosis (P=0.670), AFP level (P=0.721), Child-Pugh score (P=0.071) or SII level (P=0.438). Multivariate Cox proportional hazards regression analysis showed that first attempt incomplete response (P<0.001; HR, 2.537; 95% CI, 1.620-3.975) and Child-Pugh class B (P=0.017; HR, 1.905; 95% CI, 1.122-3.234) were independent risk factors for tumor progression. The details of univariate and multivariate Cox regression analysis for PFS are presented in **Table 3**.

**Table 3:** Univariate and multivariate Cox regression analysis for PFS.

	Univariate Cox regression		Multivariate Cox regression	
	HR (95% CI)	P	Adjusted HR (95% CI)	P
Age (>56 years)	1.018 (0.735, 1.410)	0.914	1.073 (0.760, 1.516)	0.687
Gender (male)	1.123 (0.706, 1.785)	0.624	1.228 (0.734, 2.054)	0.434
Number of tumors (multiple)	1.125 (0.794, 1.594)	0.507	1.198 (0.832, 1.726)	0.332
Modality of guidance for ablation (US)	1.178 (0.828, 1.676)	0.361	1.027 (0.713, 1.478)	0.887
Type of ablation (MWA)	0.713 (0.403, 1.260)	0.244	0.680 (0.376, 1.229)	0.202
Tumor size (>3cm)	1.102 (0.795, 1.527)	0.560	1.234 (0.839, 1.814)	0.285
First attempt ablation (incomplete response)	2.076 (1.417, 3.041)	<0.001	2.537 (1.620, 3.975)	<0.001
Etiology of hepatitis (HBV)	1.066 (0.588, 1.931)	0.834	1.039 (0.561, 1.925)	0.903
Cirrhosis (presence)	1.075 (0.769, 1.504)	0.670	1.092 (0.756, 1.578)	0.639
AFP (>400 ng/mL)	1.075 (0.722, 1.600)	0.721	1.118 (0.774, 1.613)	0.553
Child-Pugh class (B)	1.551 (0.963, 2.498)	0.071	1.905 (1.122, 3.234)	0.017
SII (≤330)	0.867 (0.606, 1.242)	0.438	0.821 (0.553, 1.218)	0.327

PFS, progression-free survival; US, ultrasound; MWA, microwave ablation; HBV, hepatitis B virus; AFP, alpha fetoprotein; SII, systemic immune inflammation index.

The univariate analysis for OS showed that a higher AFP level (P=0.019) and a first attempt incomplete response (P=0.048) were associated with poor OS. However, there were no differences between the two groups in age (P=0.512), gender (P=0.816), the number of tumors (P=0.228), modality of guidance for ablation (P=0.335), thermal ablation type (P=0.242), tumor size (P=0.256), the etiologies of hepatitis (P=0.737), presence or absence of cirrhosis (P=0.839), Child-Pugh score (P=0.089), or SII level (P=0.964). Multivariate Cox proportional hazards regression analysis showed that a higher AFP level (P=0.011; HR: 2.497; 95% CI: 1.236-5.042) and Child-Pugh class B (P=0.026; HR: 2.799; 95% CI: 1.131-6.924) were independent risk factors for poor OS. The details of univariate and multivariate Cox regression analysis for OS are presented in **Table 4**.

**Table 4:** Univariate and multivariate Cox regression analysis for OS.

	Univariate Cox regression		Multivariate Cox regression	
	HR (95% CI)	P	Adjusted HR (95% CI)	P
Age (>56 years)	1.226 (0.667, 2.252)	0.512	1.205 (0.637, 2.281)	0.566
Gender (male)	1.101 (0.489, 2.479)	0.816	1.367 (0.569, 3.288)	0.484
Number of tumors (multiple)	1.467 (0.786, 2.737)	0.228	1.782 (0.907, 3.500)	0.094
Modality of guidance for ablation (US)	1.438 (0.687, 3.009)	0.335	1.487 (0.618, 3.576)	0.461
Type of ablation (MWA)	0.615 (0.272, 1.389)	0.242	0.673 (0.280, 1.618)	0.376
Tumor size (>3cm)	1.429 (0.771, 2.646)	0.256	1.108 (0.538, 2.281)	0.781
First attempt ablation (incomplete response)	1.937 (1.006, 3.729)	0.048	1.873 (0.889, 3.947)	0.099
Etiology of hepatitis (HBV)	1.428 (0.178, 11.438)	0.737	2.355 (0.254, 21.859)	0.451
Cirrhosis (presence)	1.067 (0.569, 2.000)	0.839	1.565 (0.764, 3.205)	0.221
AFP (>400 ng/mL)	2.131 (1.130, 4.015)	0.019	2.497 (1.236, 5.042)	0.011
Child-Pugh class (B)	1.959 (0.903, 4.249)	0.089	2.799 (1.131, 6.924)	0.026
SII (≤330)	0.985 (0.511, 1.899)	0.964	0.795 (0.373, 1.695)	0.553

OS, overall survival; US, ultrasound; MWA, microwave ablation; HBV, hepatitis B virus; AFP, alpha fetoprotein; SII, systemic immune inflammation index.

## Discussion

As a curative treatment modality, thermal ablation is widely used for HCC patients (12–14). However, incomplete tumor necrosis hampers its therapeutic effect. This study aimed to investigate whether incomplete thermal ablation was associated with tumor progression and compare the efficacy of repeated thermal ablation and TACE in patients with residual tumors after an incomplete ablation. The results of this study demonstrated that the PFS rates were significantly higher in patients with first attempt complete response than those with an incomplete response, before and after a one-to-one PSM analysis.

Usually, the endpoint of thermal ablation is adequate tissue heating to induce coagulative necrosis throughout the defined target area (15). This endpoint may not easily be achieved in lesions of a large size or in unfavorable locations because heat cannot be conducted to the edge of the tumor, thereby causing reversible cellular injury and insufficient ablation. The detail mechanism of insufficient ablation resulting a tumor progression was still unknown. In several pre-clinical studies, the mechanism of the relationship between tumor progression and insufficient tumor ablation-induced reversible cellular injury has been demonstrated. Insufficient ablation exposes viable tumor to sublethal heat stress, and HCC cells may display a more malignant phenotype. Jondal et al. reported that moderate heat stress may result in insufficient ablation and stimulate HCC growth, with the mechanism partly mediated by the growth factor dependent PI3K/mTOR/AKT signaling pathway (9). Moreover, Sublethal heat stress-induced O-GlcNAcylation coordinates the Warburg effect to promote tumor progression by providing anabolic substrates to meet the high energy demand of rapid cell division (16). Several previous studies have also demonstrated insufficient thermal ablation accelerates the residual tumor progression by promoting Epithelial-Mesenchymal Transition (EMT), increasing tumor-infiltrating myeloid-derived suppressor cells (MDSCs) and reducing T-Cell-Mediated anti-tumor immune responses (17, 18). According to the results of the pre-clinical studies, patients with first attempt incomplete response are theoretically more prone to tumor progression than patients with first attempt complete response, and the results of the present clinical study support these findings. The present study also showed no difference in OS between patients with first attempt complete response and patients with incomplete response following a repeated ablation or TACE after a one-to-one PSM analysis. The probable reason for this result may be attributed to the following: (a) although patients with first attempt incomplete ablation showed a poor PFS than that of patients with complete ablation, re-treatment with repeated ablation or TACE is still effective in such patients (19, 20); (b) the OS of HCC patients is related to numerous factors, such as age, liver function, the severity of the underlying liver disease, tumor burden, antiviral therapy, and performance status, the first attempt incomplete ablation is not sufficient to influence the OS of HCC patients (21, 22).

Additionally, the multivariate Cox regression analysis showed that a first attempt incomplete response and Child-Pugh class B were independent risk factors for PFS, while a higher AFP level and Child-Pugh class B were independent risk factors for OS. These results are consistent with the aforementioned results that first attempt incomplete ablation might not influence the OS of patients, despite the poor PFS. Regarding the Child-Pugh class, it has been reported that the therapeutic outcomes of patients with HCC are associated with liver function by several previous studies (23, 24) and the present study has shown a similar result. High AFP level represents a more aggressive nature of the tumor and has been associated with a poor therapeutic outcome in patients with HCC (25, 26); the results of the present study have confirmed this finding.

The present study also compared the therapeutic outcomes between patients with repeated ablation and TACE after a failed first attempt thermal ablation. Its findings are in line with those of previous studies. A retrospective study conducted by Xie et al. (27) demonstrated that re-ablation is the first choice of treatment for patients with a local recurrence of HCC after an initial thermal ablation. A cohort study conducted by Kim et al. (28) showed that TACE is an effective treatment for patients with initial thermal ablation failure. However, previous studies focused on the treatment choice in HCC patients with local recurrence after a first attempt complete ablation, while the present study focused on the feasibility of repeated ablation or TACE for HCC patients following the first attempt incomplete ablation. Thus far, there is no standard treatment modality established for patients with residual tumor after a first attempt incomplete thermal ablation. Nonetheless, the present study compared the treatment efficacy of repeated ablation and TACE in patients with residual tumor after a first attempt thermal ablation and the results showed no significant differences in PFS and OS between the two groups. Although the present study showed a similar long-term therapeutic outcome with repeated ablation and TACE, it should be noted that thermal ablation is a curative therapy while TACE is a palliative therapy, and TACE should be performed as an alternative option when ablation is not feasible.

There are several limitations to the present study. First, this was a multicenter and retrospective study with an inevitable heterogeneity in treatments (two centers, different ablation devices) between the institutions, which may have led to a selection bias. Although propensity score-matching (PSM) was applied to diminish potential confounding and selection bias, there were still some heterogeneous between the two groups, such as follow up imagine (CT/MRI) and ablation type (MWA/RFA). Second, the limited follow-up period of the study may have impeded the thorough survival assessment of the patients. Third, the limited number of patients with first attempt incomplete ablation who received repeated ablation (n=29) or TACE (n=19) may hamper the reliability of the results. The long-term therapeutic outcomes of the two groups need to be investigated in further studies.

In conclusion, although patients with an incomplete response after a first attempt thermal ablation were more prone to tumor progression than those with a first attempt complete ablation, their OS was not affected by incomplete ablation. Besides, when patients present with a residual tumor after first attempt thermal ablation, TACE may be considered an alternative option if repeated ablation is not feasible in them.

## Data Availability Statement

The raw data supporting the conclusions of this article will be made available by the authors, without undue reservation.

## Ethics Statement

The studies involving human participants were reviewed and approved by Second Xiangya Hospital and the Affiliated Cancer Hospital of Xiangya School of Medicine.

## Author Contributions

JT and Y-DX analyze and interpret the patients’ data and review the patients’ image. TT and Z-SZ provide the patients data. JT, WZ, and Y-DX collect the data and revise the manuscript. Y-DX, WZ, and JT are three major contributors in writing the manuscript. Y-DX provide the concept, and Y-DX is a major contributor in the manuscript editing. All authors read and approve the final manuscript.

## Funding

Project supported by the National Nature Science Foundation of China (Grant No. 82102157), Natural Science Foundation of Hunan Province (Grant No. 2021JJ40895; 2021JJ30964), Science and Technology Program of Hunan Province (Grant No. 2020SK53423; 2020SK53407). The funder had no role in study design, data collection and analysis, decision to publish, or manuscript preparation.

## Conflict of Interest

The authors declare that the research was conducted in the absence of any commercial or financial relationships that could be construed as a potential conflict of interest.

## Publisher’s Note

All claims expressed in this article are solely those of the authors and do not necessarily represent those of their affiliated organizations, or those of the publisher, the editors and the reviewers. Any product that may be evaluated in this article, or claim that may be made by its manufacturer, is not guaranteed or endorsed by the publisher.
